# Fragility Fractures and Cortisol Secretion in Patients With Nonfunctioning Adrenal Incidentalomas

**DOI:** 10.1210/jendso/bvae144

**Published:** 2024-08-16

**Authors:** Vittoria Favero, Elisa Cairoli, Cristina Eller-Vainicher, Valentina Morelli, Antonio Stefano Salcuni, Silvia Della Casa, Giovanna Muscogiuri, Carla Columbu, Flavia Pugliese, Sabrina Corbetta, Luca Persani, Alfredo Scillitani, Iacopo Chiodini

**Affiliations:** Department of Medical Biotechnology and Translational Medicine, University of Milan, 20100 Milan, Italy; UOSD Bone Metabolic Diseases and Diabetes, Department of Endocrine and Metabolic Diseases, IRCCS Istituto Auxologico Italiano, 20100 Milan, Italy; Unit of Endocrinology, Fondazione IRCCS Cà Granda-Ospedale Maggiore Policlinico Milan, 20100 Milan, Italy; UOSD Bone Metabolic Diseases and Diabetes, Department of Endocrine and Metabolic Diseases, IRCCS Istituto Auxologico Italiano, 20100 Milan, Italy; Unit of Endocrinology and Metabolism, University Hospital S. Maria Della Misericordia, 33100 Udine, Italy; Department of Medical and Surgical Sciences, Fondazione Policlinico Universitario A. Gemelli, 00100 Rome, Italy; Dipartimento di Medicina Clinica e Chirurgia, Unità di Endocrinologia, Andrologia e Diabetologia—Università Federico II di Napoli, 80110 Naples, Italy; UNESCO Chair “Education for Health and Sustainable Development,” University of Naples “Federico II,” 80131 Naples, Italy; Unit of Endocrinology “Casa Sollievo della Sofferenza,” Hospital, IRCCS, San Giovanni Rotondo, 71013 Foggia, Italy; Unit of Endocrinology “Casa Sollievo della Sofferenza,” Hospital, IRCCS, San Giovanni Rotondo, 71013 Foggia, Italy; UOSD Bone Metabolic Diseases and Diabetes, Department of Endocrine and Metabolic Diseases, IRCCS Istituto Auxologico Italiano, 20100 Milan, Italy; Department of Biomedical, Surgical and Dental Sciences, University of Milan, 20100 Milan, Italy; Department of Medical Biotechnology and Translational Medicine, University of Milan, 20100 Milan, Italy; UOSD Bone Metabolic Diseases and Diabetes, Department of Endocrine and Metabolic Diseases, IRCCS Istituto Auxologico Italiano, 20100 Milan, Italy; Unit of Endocrinology “Casa Sollievo della Sofferenza,” Hospital, IRCCS, San Giovanni Rotondo, 71013 Foggia, Italy; Department of Medical Biotechnology and Translational Medicine, University of Milan, 20100 Milan, Italy; Unit of Endocrinology, ASST Grande Ospedale Metropolitano Niguarda, 20162 Milan, Italy

**Keywords:** adrenal incidentaloma, fractures, cortisol, bone density, osteoporosis

## Abstract

**Context:**

The risk of vertebral fractures (VFx) in patients with nonfunctioning adrenal incidentalomas (NFAI) is unknown.

**Objective:**

This work aimed to assess in NFAI patients the prevalence and incidence of VFx and a hormonal marker to identify patients at risk.

**Methods:**

A retrospective, cross-sectional, and longitudinal study of outpatients was conducted. A total of 306 NFAI patients (cross-sectional arm) and 213 controls were evaluated for VFx prevalence; 85 NFAI patients (longitudinal arm, follow-up 30.3 ± 17.5 months) were evaluated for VFx incidence. Main outcome measures included serum cortisol after 1 mg-dexamethasone test (F-1mgDST), lumbar spinal (LS), and femoral neck (FN) bone mineral density (BMD) and VFx presence, by radiograph of the spine.

**Results:**

Cross-sectional arm: prevalent VFx associated with F-1mgDST with a cutoff of 1.2 µg/dL (33 nmol/L, area under the curve 0.620 ± 0.39; *P* = .002). Compared with controls and NFAI patients with F-1mgDST less than 1.2 µg/dL (group A), NFAI patients with F-1mgDST greater than or equal to 1.2 µg/dL (group B) showed a higher VFx prevalence (10.8%, 12.6%, and 29.5%, respectively; *P* < .001 all comparisons), which was associated with F-1mgDST greater than or equal to 1.2 µg/dL (odds ratio 3.02; 95% CI, 1.63-5.58; *P* < .001) accounting to confounders. Longitudinal arm: the VFx incidence was higher in group B than in group A (19.3% vs 3.6%; *P* = .05). In group B, all incident VFx occurred in patients without low BMD. The F-1mgDST cutoff for predicting an incident VFx was 1.2 µg/dL, although statistical significance was not reached after adjustment for confounders (*P* = .061).

**Conclusion:**

In NFAI patients, F-1mgDST levels greater than or equal to 1.2 µg/L (33 nmol/L) are associated with prevalent VFx and may identify patients at risk of incident VFx.

In the recent years, the condition of mild autonomous cortisol secretion (MACS) in patients with adrenal incidentalomas (AIs) has received particular attention, since MACS has been associated with several comorbidities, increased cardiovascular risk, and mortality [1-5]. More recently, MACS has been suggested to be associated also with an increased risk of vertebral fracture (VFx) [6, 7]. Above all, the importance of diagnosing MACS is that in AI patients the MACS-associated comorbidities (ie, hypertension, diabetes mellitus, and bone fragility) are potentially reversible after the removal of the adrenal mass [8-10].

Although, nowadays, the MACS condition is clearly defined [3], it is also well known that in AI the cortisol secretion is a “continuum” from normal cortisol levels to a clear cortisol excess [11], this concept explaining why even some patients with so-called “nonfunctioning” AI (NFAI) are at higher risk of cardiovascular events [11-14]. In keeping with this idea, a not negligible percentage of patients with AI but without MACS, who undergo unilateral adrenalectomy, may have postsurgical hypocortisolism [15]. Interestingly, the cortisol levels after a 1-mg overnight dexamethasone suppression test (F-1mgDST) before surgery with a cutoff of 1.8 µg/dL (50 nmol/L) as suggested by current guidelines [3] do not completely rule out the risk of postoperative hypocortisolism [15]. Moreover, very recently, in a large sample of NFAI patients, the presence of hypertension and/or diabetes mellitus was associated with F-1mgDST levels greater than or equal to 1.2 µg/dL (33 nmol/L), substantially confirming previous data showing that the cutoff of F-1mgDST with the best accuracy for predicting cardiovascular risk [11, 16] and incidence of diabetes mellitus [12] in AI patients was below 1.5 µg/dL (41 nmol/L). Finally, some data suggest that the mortality risk associated with NFAI is similar to that in AI patients with MACS [4] and that adrenalectomy may improve blood pressure and diabetes control even in patients with NFAI [10]. These findings suggest that a certain degree of cortisol hypersecretion might be present in some individuals with AI but without biochemical evidence of cortisol hypersecretion.

While cardiovascular risk is starting to be investigated even in the condition of NFAI, no data are available on the effect of a possible degree of cortisol hypersecretion on bone fragility in patients with NFAI. Therefore, the aim of the present study was to evaluate, in a sample of NFAI patients, the bone mineral density (BMD) and the prevalence and incidence of VFx and their possible association with the degree of cortisol secretion.

## Materials and Methods

### Study Design

In the present study, we analyzed data of NFAI patients collected from January 1997 to June 2013, for a previous retrospective study, performed in 4 referral Italian Hospitals (Scientific Institute “Casa Sollievo della Sofferenza” in San Giovanni Rotondo, “San Giuseppe” Hospital in Milan, Scientific Institute “Fondazione Cà Granda-Ospedale Maggiore Policlinico” in Milan, and Scientific Institute “Policlinico San Donato” in San Donato Milanese) and aimed to evaluate if the indexes of hypothalamic-pituitary-adrenal (HPA) axis activity could be used for identifying AI patients at risk of VFx [17]. In the present study, we added data of NFAI patients collected from July 2013 to December 2023, at the Scientific Institute “Casa Sollievo della Sofferenza” in San Giovanni Rotondo. We evaluated for study inclusion all NFAI patients for whom data about BMD, VFx, and HPA axis activity were available.

In the cross-sectional arm of the study, we compared data from 306 NFAI patients (34 premenopausal eugonadal women, 153 postmenopausal women, 119 eugonadal men) with those of 213 individuals (16 premenopausal eugonadal women, 107 postmenopausal women, 90 eugonadal men) matched for age, sex, and body mass index (BMI) recruited as a control group, to assess the association between VFx prevalence and presence of NFAI. We then evaluated the independent association between VFx prevalence and the parameters of HPA axis activity and their best cutoff for individuating NFAI patients with VFx.

In the longitudinal arm of the study, we enrolled 85 NFAI patients (37 postmenopausal women, 20 premenopausal women, 25 eugonadal men) for whom follow-up data (24 months at least) were available, for assessing the independent association between VFx incidence and the parameters of HPA axis activity.

### Participants

The inclusion criteria were (i) the presence of AI incidentally found by abdominal imaging, performed for unrelated diseases, and confirmed with unenhanced computed tomography; (ii) tumor size greater than 1 cm; (iii) well-shaped tumor features consistent with the diagnosis of adrenocortical adenoma (ie, hypodense, homogeneous, and with Hounsfield units <10).

The exclusion criteria were (i) clinically evident cortisol excess (ie, presence of striae rubrae, moon facies, buffalo hump, proximal muscle weakness, and skin atrophy) and/or presence of MACS (ie, F-1mgDST levels >1.8 µg/dL, 50 nmol/L); (ii) history of hypogonadism (in men testosterone levels <300 ng/dL, 10.4 nmol/L, and in premenopausal women <6 menstrual cycles/year), chronic hepatic or kidney disease, eating disorder, thyrotoxicosis, bowel diseases, rheumatologic or hematological diseases, and alcoholism; (iii) use of drugs influencing cortisol metabolism (ie, oral contraceptives for premenopausal women and gonadal hormone replacement therapy for postmenopausal women) or secretion or dexamethasone metabolism; (iv) presence of bilateral adrenal masses; (v) presence of metastatic diseases; and (vi) coexistent hyperaldosteronism.

The individuals who served as controls were enrolled during the same period of time on the basis of the aforementioned inclusion criteria from our outpatient clinics at which they were referred for multinodular goiter with normal thyroid function. All control individuals with osteoporosis or low BMD underwent F-1mgDST, and none of the included participants had F-1mgDST greater than 1.8 g/dL (50 nmol/L).

### Materials and Methods

All patients with hypovitaminosis D were supplemented with cholecalciferol by mouth to achieve normal vitamin D levels (ie, >30 ng/mL, 75 nmol/L). All patients with insufficient dietary calcium intake (ie, <1000 mg/d) were supplemented with oral calcium carbonate or calcium citrate, as appropriate.

In all patients, within 3 months of discovering AI, morning plasma adrenocorticotropin (ACTH) levels (normal values 10-55 pg/mL, 2.2-12 pmol/L), 24-hour urinary cortisol (UFC) levels (normal values, 10-70 µg/24 hours, 28-193 nmol/24 hours) and, on a different day, F-1mgDST were been measured. Plasma ACTH levels were measured by immunoradiometric assay (BRAHMS Diagnostica GmbH) and reported as the mean of 3 determinations at 20-minute intervals. Serum cortisol and UFC levels (after dichloromethane extraction) were measured immunofluorometrically by TDxFLx kits (Abbott GmbH Diagnostika). The intra-assay and interassay coefficients of variation were less than 15% for ACTH and less than 10% for cortisol. In both the cross-sectional and longitudinal arm, the reported HPA axis parameters were those determined at baseline.

BMD was measured in all patients and controls by dual-energy x-ray absorptiometry (Hologic Discovery, software version 13.3:3) at both the femoral neck (FN, precision 1.8%) and lumbar spine (LS, precision 1.0%) and reported as SD units (Z-score). A thoracolumbar (T4-L4) spinal radiograph in lateral and anteroposterior projection was performed on all participants and reviewed by a trained radiologist, who was blinded to BMD and hormonal data. The semiquantitative visual assessment was used to diagnose prevalent and incident VFx [18]. Based on the commonly used criteria, the presence of osteoporosis was defined by a BMD T-score less than or equal to −2.5 at any skeletal site and/or the presence of a fragility fracture either clinically or determined on the x-ray of the spine [19]. In the absence of a fragility fracture, the term “*low BMD*” was used to define individuals with a T-score at any site less than or equal to −2.5 for postmenopausal women and men older than 50 or with a Z-score at any site less than or equal to −2.0 for premenopausal women and men younger than 50 years [20].

Finally, in all patients the BMI was calculated, and the presence of type 2 diabetes mellitus (T2D) was recorded [21].

All participants signed an informed consent before entering the study. The protocol has been approved by the ethics committees of the participating centers.

### Statistical Analysis

Statistical analysis was performed by GraphPad Prism version 9 (GraphPad Software) and SPSS version 28.0 statistical package (IBM Corporation). Results are expressed as mean ± SD, if not differently specified. The normality of the data distribution was tested using the Kolmogorov-Smirnov test.

First, the receiver operating characteristic (ROC) curve was used to evaluate the association between either F-1mgDST or ACTH or UFC levels and the prevalence of VFx. Then, for statistically significant associations, the ROC curve identified the F-1mgDST and/or ACTH and/or UFC cutoffs and their areas under the curve with its 95% CI with the best diagnostic accuracy in individuating NFAI patients with VFx. The Youden index (J = sensitivity + specificity − 1) was used to identify the most appropriate cutoff.

Subsequently, we planned to compare the clinical characteristics of patients subdivided on the basis of the F-1mgDST and/or ACTH and/or UFC cutoffs identified by the ROC curve. Comparison of continuous variables was performed using the *t* test or Mann-Whitney *U* test, as appropriate. One-way analysis of variance test with post hoc Bonferroni comparison test was performed to analyze the differences between controls and the 2 groups of patients subdivided on the basis of the cutoffs of F-1mgDST and/or ACTH and/or UFC levels as determined by the ROC curve analysis. In the same groups, the categorical variables were compared using the χ^2^ test or Fisher exact test, as appropriate.

The logistic regression analysis was used to assess independent associations between the presence of prevalent VFx (in the cross-sectional arm) or of incident VFx (in the longitudinal arm) after adjusting for the variables that were different between patients stratified on the basis of the F-1mgDST or ACTH or UFC cutoffs (defined by the ROC curve analysis) and for the possible influencing factors, such as age, BMI, sex, postmenopausal status, BMD, and presence of T2D, which is known to possibly increase VFx risk regardless of BMD [22]. The presence of prevalent VFx was included among the covariates possibly predicting the presence of incident VFx in the longitudinal arm, since it is known that a previous fragility fracture increases the risk of subsequent fractures [23].

*P* values of less than .05 were considered statistically significant.

## Results

### Cross-Sectional Arm: All Participants and Controls

Age, proportion of female sex, of premenopausal status, and of nonvertebral Fx, BMI, and BMD at both the LS and FN were comparable between NFAI patients and controls. The prevalence of individuals with VFx, low BMD, osteoporosis, and T2D was higher in NFAI patients than in controls; however, it did not reach statistical significance for T2D (Table 1).

**Table 1.** Cross-sectional arm: characteristics of patients with nonfunctioning adrenal incidentalomas and of control individuals

	Controls (n = 213)	NFAI (n = 306)	*P*
Age, y	59.9 ± 12.3 (21 to 79)	60.6 ± 12.0 (20 to 89)	.509
Women	123 (57.7)	187 (61.1)	.442
BMI	30.2 ± 4.9 (20.5 to 42.6)	30.0 ± 5.1 (19.5 to 43.0)	.593
Premenopausal women	16 (13.0)	34 (18.2)	.226
Type 2 diabetes mellitus	28 (13.1)	59 (19.3)	.066
Low BMD	30 (14.1)	71 (23.7)	.**007**
Osteoporosis	36 (16.9)	87 (29.1)	.**001**
Prevalent VFx	23 (10.8)	61 (19.9)	.**005**
Nonvertebral Fx	5 (2.3)	15 (4.9)	.137
LS BMD (Z-score)	0.16 ± 0.99 (−1.90 to 2.86)	0.14 ± 1.30 (−3.60 to 4.1)	.851
FN BMD (Z-score)	0.09 ± 0.93 (−2.30 to 2.88)	0.03 ± 0.96 (−2.80 to 4.30)	.483

Categorical variables are reported as absolute number with percentage in parentheses. Continuous variables are reported as mean ± SD with range in parentheses. Statistically significant comparisons are in bold. Osteoporosis: BMD T-score less than or equal to −2.5 at any skeletal site and/or presence of a fragility fracture. Low BMD: T-score at any site less than or equal to −2.5 for postmenopausal women and men older than 50 years or with Z-score at any site less than −2.0 for premenopausal women and men younger than 50 years.

Abbreviations: BMD, bone mineral density; BMI, body mass index; FN, femoral neck; Fx, fractures; NFAI, nonfunctioning adrenal incidentaloma; Nonvertebral Fx, fragility fractures in other skeletal sites (femur, humerus, and wrist); LS, lumbar spine; VFx, vertebral fractures.

The ROC curve analysis showed that the presence of a prevalent VFx was associated with F-1mgDST levels (area under the curve 0.620 ± 0.39; *P* = .002), but not with ACTH or UFC levels. The cutoff with the best accuracy for identifying patients with a prevalent VFx was found to be 1.2 µg/dL (33 nmol/L).

We then compared the clinical characteristics of patients with F-1mgDST less than 1.2 µg/dL (33 nmol/L, group A) with those of patients with F-1mgDST greater than or equal to 1.2 µg/dL (33-49.4 nmol/L) (group B).

**Figure 1.**
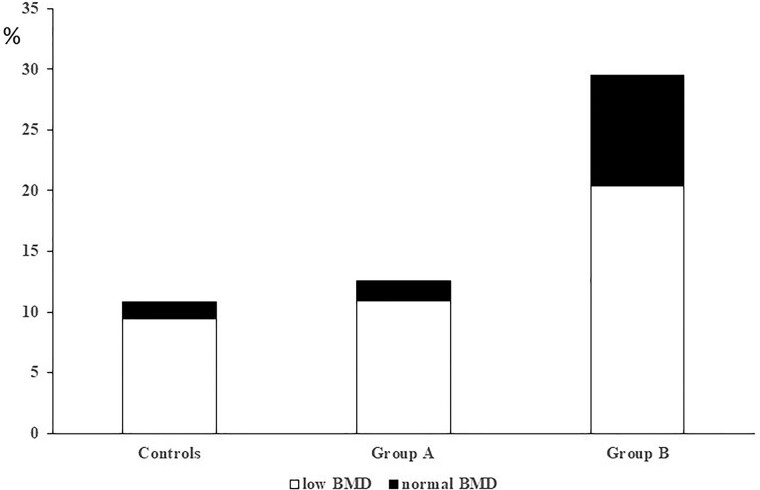
Prevalence of vertebral fractures (VFx) at baseline in relation with the presence of low bone mineral density (BMD) in control individuals, in patients from group A and from group B. Low BMD: presence of T-score at any site less than or equal to −2.5 for postmenopausal women and men older than 50 years or of Z-score at any site less than or equal to −2.0 for premenopausal women and men younger than 50 years. Normal BMD: presence of T-score at any site greater than −2.5 for postmenopausal women and men older than 50 years or of Z-score at any site greater than −2.0 for premenopausal women and men younger than 50 years. Controls: age-, sex-, and body mass index–matched control individuals. A, Patients with nonfunctioning adrenal adenoma (NFAI) with cortisol after 1-mg overnight dexamethasone suppression test (F-1mgDST) less than 1.2 µg/dL (33 nmol/L). B, NFAI patients with F-1mgDST greater than or equal to 1.2 µg/dL (33 nmol/L). VFx prevalence was higher in B patients (29.5%) compared both with controls and A patients (10.8% and 12.6%, respectively; *P* < .001 for both comparisons). VFx prevalence in the absence of low BMD was higher in B patients (35.6%) compared both with controls and A patients (13.0% and 13.6%, respectively; *P* < .001 for both comparisons).

As shown in Table 2, the patients in group B were older and had larger tumor size than those in group A. Moreover, group B patients showed a higher percentage of T2D, low BMD, osteoporosis, VFx, and nonvertebral VFx and of VFx without low BMD (Fig. 1) compared both to group A patients and controls. No differences were found in the prevalence of female sex and premenopausal status, BMI, and of ACTH and UFC levels between A, B, and the control groups.

**Table 2.** Cross-sectional arm: characteristics of patients with nonfunctioning adrenal incidentalomas stratified based on 1-mg overnight dexamethasone suppression test less than 1.2 µg/dL (group A) or greater than or equal to 1.2 µg/dL (group B) and of control individuals

	Controls (n = 213)	Group A (n = 174)	Group B (n = 132)	*P*
Age, y	59.9 ± 12.3 (21 to 79)	59.1 ± 11.8 (20 to 78)	62.5 ± 12.1*^a^* (21 to 89)	.046
Women	123 (57.7)	110 (63.2)	77 (58.3)	.513
BMI	30.2 ± 4.9 (20.5 to 42.6)	30.3 ± 5.2 (20.0 ‒43.0)	29.6 ± 4.9 (19.5 to 40.8)	.501
Premenopausal women	16 (13.0)	25 (22.7)	9 (11.7)	.721
Tumor size, cm	‒	2.1 ± 0.9 (1.0 to 6.0)	2.3 ± 0.8*^a^* (1.0 to 5.0)	.**045**
F-1mgDST, µg/dL	‒	0.86 ± 0.22 (0.50 to 1.19)	1.48 ± 0.20*^b^* (1.20 to 1.80)	**<**.**001**
UFC, µg/24 h	‒	47.6 ± 28.0 (10.0 to 169.1)	46.8 ± 24.0 (10.0 to 120.0)	.805
ACTH, pg/mL	­‒	15.7 ± 8.7 (3.8 to 48.3)	16.6 ± 9.5 (2.8 to 48.3)	.402
Type 2 diabetes mellitus	28 (13.1)	22 (12.6)	37*^c^* (28.0)	**<**.**001**
Low BMD	30 (14.1)	33 (19.0)	38*^c^* (28.4)	**<**.**001**
Osteoporosis	36 (16.9)	38 (21.8)	49*^c^* (37.1)	**<**.**001**
Prevalent VFx	23 (10.8)	22 (12.6)	39*^c^* (29.5)	**<**.**001**
VFx without low BMD*^e^*	3 (13.0)	3 (13.6)	14*^d^* (35.9)	.**05**
Nonvertebral Fx	5 (2.3)	3 (1.7)	12*^d^* (9.1)	**<**.**001**
LS BMD (Z-score)	0.16 ± 0.99 (−1.90 to 2.86)	0.18 ± 1.18 (−2.81 to 3.61)	0.09 ± 1.48 (−3.60 to 4.10)	.796
FN BMD (Z-score)	0.09 ± 0.93 (−2.30 to 2.88)	0.02 ± 0.92 (−2.80 to 2.40)	0.05 ± 1.03 (−1.70 to 4.30)	.740

Categorical variables are reported as absolute number with percentage in parentheses. Continuous variables are reported as mean ± SD with range in parentheses. Statistically significant comparisons are in bold. Osteoporosis: BMD T-score less than −2.5 at any skeletal site and/or presence of a fragility fracture. Low BMD: T-score at any site less than or equal to −2.5 for postmenopausal women and men older than 50 or with Z-score at any site less than −2.0 for premenopausal women and men younger than 50 years.

Abbreviations: ACTH, adrenocorticotropin; BMD, bone mineral density; BMI, body mass index; F-1mgDST: 1-mg overnight dexamethasone suppression test; FN, femoral neck; Fx, fractures; LS, lumbar spine; Nonvertebral Fx, fragility fractures in other skeletal sites (femur, humerus, and wrist); UFC, urinary free cortisol; VFx, vertebral fractures.

^*a*^ *P* less than .05.

^*b*^ *P* less than .05 vs group A.

^*c*^ *P* less than .001.

^*d*^ *P* less than .01.

^*e*^Percentage refers to patients with VFx.

Logistic regression analysis showed that the presence of a prevalent VFx was independently associated with the presence of F-1mg DST greater than or equal to 1.2 µg/dL (33 nmol/L), age, and LS BMD but not with BMI, presence of T2D, and sex (Table 3).

**Table 3.** Cross-sectional arm: independent associations between presence of a prevalent vertebral fragility fracture and presence of 1-mg overnight dexamethasone suppression test greater than or equal to 1.2 µg/dL, age, body mass index, sex, presence of type 2 diabetes mellitus, and lumbar spine bone mineral density in patients with nonfunctioning adrenal incidentalomas

	OR	95% CI	*P*
Group B, presence	3.02	1.63-5.58	**<**.**001**
Age, 1-y increase	1.03	1.01-1.06	.**043**
BMI, 1-unit decrease	1.02	0.96-1.09	.459
Sex, women	1.39	0.74-2.61	.312
LS-BMD, 1 Z-score decrease	1.35	1.72-10.5	.017
Type 2 diabetes mellitus, absence	1.37	0.61-3.09	.443

Group B: NFAI patients with cortisol after F-1mg DST greater than or equal to 1.2 µg/dL (33 nmol/L). Statistically significant associations are in bold.

Abbreviations: BMD, bone mineral density; BMI, body mass index; F-1mg DST, 1-mg overnight dexamethasone suppression test; LS, lumbar spine; NFAI, nonfunctioning adrenal incidentaloma; OR, odds ratio.

Overall, these results were substantially confirmed even when men and women were separately evaluated. The comparisons of men or women subdivided in group A and group B and their respective controls are detailed in Supplementary Tables S1 and S2 [24], respectively.

### Longitudinal Arm: All Participants

**Figure 2.**
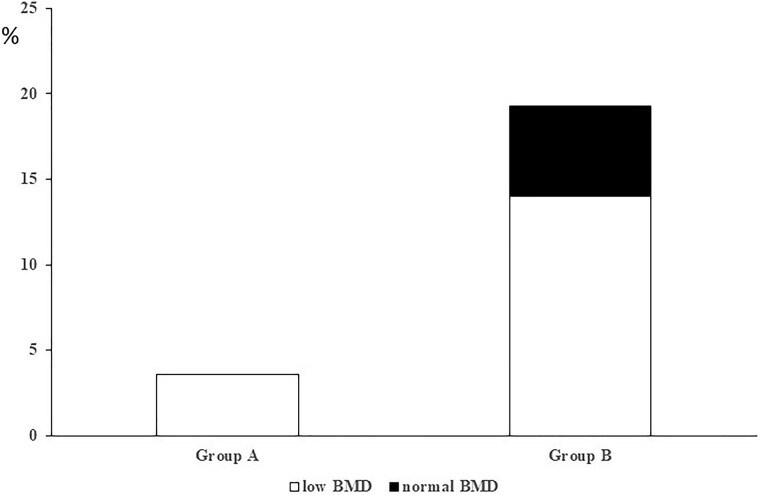
Incidence of vertebral fractures (VFx) in relation with the presence of low bone mineral density (BMD) in a subgroup of patients from group A and from group B. Low BMD: presence of T-score at any site less than or equal to −2.5 for postmenopausal women and men older than 50 or of Z-score at any site less than or equal to −2.0 for premenopausal women and men younger than 50 years. Normal BMD: presence of T-score at any site greater than −2.5 for postmenopausal women and men older than 50 years or of Z-score at any site greater than −2.0 for premenopausal women and men younger than 50 years. A, Patients with nonfunctioning adrenal adenoma (NFAI) with cortisol after 1-mg overnight dexamethasone suppression test (F-1mgDST) less than 1.2 µg/dL (33 nmol/L). B, NFAI patients with F-1mgDST greater than or equal to 1.2 µg/dL (33 nmol/L). VFx incidence was higher in B patients (19.3%) compared with A patients (3.6%; *P* < .05). In A patients all incident VFx occurred in the presence of low BMD, while in B patients the 36.4% of incident VFx occurred in the absence of low BMD.

Data from the longitudinal arm of the study are reported in Table 4. Age was higher in group B patients than in group A. The proportion of women and of premenopausal women, duration of follow-up, tumor size, and prevalence of T2D were similar between the two groups. According to the hormonal pattern, group B patients had significantly higher F-1mg DST levels compared to group A patients, while ACTH levels were similar between the two groups. However, UFC levels were significantly higher in group B than in group A. No statistically significant differences were found in the prevalence of osteoporosis, prevalent VFx, and BMD values both at the LS and FN between the two groups, but the prevalence of low BMD was higher in group B than in group A. Incident VFx were higher in group B, which had 11 new VFx, compared with only 1 in group A. Importantly, in group B, all incident VFx and all nonvertebral Fx occurred in patients without low BMD (Fig. 2).

**Table 4.** Longitudinal arm: characteristics of patients with nonfunctioning adrenal incidentalomas stratified based on 1-mg overnight dexamethasone suppression test less than 1.2 µg/dL (group A) or greater than or equal to 1.2 µg/dL (group B)

	Total (n = 85)	Group A (n = 28)	Group B (n = 57)	*P*
Age, y	62.3 ± 10.9 (27 to82)	58.0 ± 11.1 (27 to 77)	64.4 ± 10.2 (41 to 82)	.**011**
Women	60 (70.6)	19 (67.9)	41 (71.9)	.699
BMI	27.6 ± 4.6 (20.0 to 41.0)	29.0 ± 4.7 (20.0 to 40.0)	26.9 ± 4.5 (20.0 to 41.0)	.060
Premenopausal women	9 (15.0)	5 (26.3)	4 (9.8)	.095
Tumor size, cm	1.9 ± 0.7 (1.0 to 3.7)	1.9 ± 0.7 (1.0 to 3.7)	2.0 ± 0.6 (1 to 3.5)	.539
Follow-up, mo	30.3 ± 17.5 (24 to 120)	27.0 ± 8.6 (24 to 60)	31.9 ± 20.4 (24 to 120)	.228
F-1mgDST, µg/dL	1.26 ± 0.40 (0.50 to 1.80)	0.80 ± 0.22 (0.50 to 1.19)	1.48 ± 0.19 (1.20 to 1.80)	**<**.**001**
UFC, µg/24 h	43.2 ± 23.0 (10.0 to 119.4)	35.8 ± 21.9 (10.0 to 102.0)	46.9 ± 22.8 (11.0 to 119.4)	.**040**
ACTH, pg/mL	16.1 ± 8.7 (5.0 to 35.0)	18.2 ± 9.4 (5.0 to 35.0)	15.1 ± 8.2 (5.8 to 35.0)	.131
Type 2 diabetes mellitus	16 (18.8)	2 (7.1)	14 (24.6)	.053
Low BMD	18 (21.2)	2 (7.1)	16 (28.1)	.**045**
Osteoporosis	25 (30.1)	5 (18.5)	20 (35.7)	.110
Prevalent VFx	19 (22.4)	7 (25.0)	12 (21.1)	.440
Incident VFx	12 (14.1)	1 (3.6)	11 (19.3)	.**050**
Incident VFx without low BMD*^a^*	4 (33.3)	0 (0.0)	4 (36.4)	.460
Nonvertebral Fx	4 (4.7)	0 (0.0)	4 (7.0)	.297
LS BMD, Z-score	−0.14 ± 1.19 (−2.03 to 4.10)	0.04 ± 0.99 (−1.60 to 2.30)	0.19 ± 1.28 (−2.03 to 4.10)	.589
FN BMD, Z-score	−0.01 ± 0.78 (−1.6 to 2.1)	−0.05 ± 0.59 (−1.2 to 1.1)	0.01 ± 0.85 (−1.6 to 2.1)	.748

Categorical variables are reported as absolute number with percentage in parentheses. Continuous variables are reported as mean ± SD with range in parentheses. Statistically significant comparisons are in bold. Osteoporosis: BMD T-score less than −2.5 at any skeletal site and/or presence of a fragility fracture. Low BMD: T-score at any site less than or equal to −2.5 for postmenopausal women and men older than 50 or with Z-score at any site less than −2.0 for premenopausal women and men younger than 50 years.

Abbreviations: ACTH, adrenocorticotropin; BMD, bone mineral density; BMI, body mass index; F-1mgDST, 1-mg overnight dexamethasone suppression test; FN, femoral neck; LS, lumbar spine; UFC, urinary free cortisol; VFx, vertebral fractures.

^*a*^Percentage refers to patients with VFx.

The ROC curve analysis showed that the presence of incident VFx tended to be associated with F-1mgDST levels (area under the curve 0.634 ± 0.86; *P* = .1), but not with ACTH or UFC levels. The cutoff with the best accuracy for identifying patients with incident VFx was confirmed to be 1.2 µg/dL (33 nmol/L).

Logistic regression analysis showed that the occurrence of incident VFx tended to be independently associated with the presence of group B, with prevalent VFx and with LS-BMD after adjusting for age, BMI, postmenopausal status, presence of T2D, and duration of follow-up (Table 5) even though statistical significance was not reached, probably due to the small sample size.

**Table 5.** Longitudinal arm: independent associations between presence of an incident vertebral fragility fracture and presence of 1-mg overnight dexamethasone suppression test greater than or equal to 1.2 µg/dL, age, body mass index, sex, postmenopausal status, presence of type 2 diabetes mellitus, and lumbar spine bone mineral density in patients with nonfunctioning adrenal incidentalomas

	OR	95% CI	*P*
Group B, presence	8.31	0.91-76.31	.061
Age, 1-y decrease	1.02	0.94-1.10	.691
BMI, 1-unit increase	1.06	0.89-1.25	.493
Postmenopausal status, presence	2.77	0.47-16.47	.262
Type 2 diabetes mellitus, presence	1.73	0.29-10.14	.541
LS-BMD, 1 Z-score decrease	2.12	0.94-4.76	.069
Patients with prevalent VFx, presence	4.56	0.89-23.35	.068
Duration of follow-up, 1-y increase	1.01	0.97-1.05	.522

Group B: NFAI patients with cortisol after F-1mg DST greater than or equal to 1.2 µg/dL (33 nmol/L).

Abbreviations: BMD, bone mineral density; BMI, body mass index; F-1mg DST, 1-mg overnight dexamethasone suppression test; LS, lumbar spine; NFAI, nonfunctioning adrenal incidentalomas; OR, odds ratio; VFx, vertebral fractures.

## Discussion

The present study shows that patients with NFAI and F-1mgDST levels greater than or equal to 1.2 µg/dL (≥33 nmol/L) have an increased prevalence of fragility fractures (both VFx and nonvertebral fractures) and of low BMD compared to age-, sex-, and BMI-matched controls and to NFAI patients with F-1mgDST levels less than 1.2 µg/dL (<33 nmol/L). Moreover, the increased VFx prevalence in patients included in group B was independent of possible influencing factors including age, sex, BMD, and presence of T2D. Importantly, in the subgroup of NFAI patients followed up for at least 2 years, the VFx incidence was associated with F-1mgDST, with the best cutoff being confirmed to be set at 1.2 µg/dL (33 nmol), even though statistical significance was not reached after adjusting for possible influencing factors. Finally, both prevalent and incident VFx were more frequent in patients without low BMD.

The idea that a certain degree of cortisol hypersecretion could be present even in some patients with AI but without MACS is supported by previous evidence. Indeed, it was reported that removal of the adrenal mass led to the amelioration of T2D and hypertension in some patients with NFAI [10]. Moreover, T2D incidence has been found to be increased in patients with NFAI compared with individuals without adrenal masses [25], and patients with NFAI and MACS have been suggested to have a similarly increased mortality risk. Likewise, some evidence exists that in AI patients the prevalence of cardiovascular events progressively increases with increasing F-1mgDST levels starting from more than 1.5 μg/dL (41 nmol/L) [26], therefore lower than the generally validated F-1mgDST cutoff (ie, 1.8 μg/dL, 50 nmol/L) for diagnosing MACS [3]. It has also been suggested that up to 29% of patients with NFAI may experience postsurgical hypocortisolism [15, 27], a finding that reinforces the idea that, in some patients with NFAI, before surgery a certain degree of cortisol excess is able to partially suppress ACTH secretion. Finally, recent data have shown that in NFAI patients, the presence of F-1mgDST levels greater than or equal to 1.2 μg/dL (33 nmol/L) was associated with a higher prevalence of hypertension and T2D and with a worse cardiometabolic profile [28].

Thus, even considering these previous data, the present study is of importance since it shows for the first time that NFAI patients with F-1mgDST greater than or equal to 1.2 μg/dL (33 nmol/L) have a significantly increased risk of VFx, therefore suggesting that it might be worth evaluating NFAI patients with F-1mgDST greater than or equal to 1.2 μg/dL (33 nmol/L) even as far as bone health is concerned. Importantly, as in patients with Cushing syndrome and in patients with MACS, the present data show that even in NFAI patients the presence of VFx is partially independent of the BMD, as suggested by the finding that a larger proportion of group B individuals had a VFx at baseline despite a normal or only slightly reduced BMD. This finding is in accordance with the glucocorticoid-related pathogenesis of bone fragility, since it is known that glucocorticoids particularly affect bone quality, lowering the predictive value of the BMD determination on fracture risk [29-32]. Interestingly, the difference in terms of cortisol secretion between group A and group B patients is in line with the findings that the latter had larger adenomas, a higher prevalence of T2D and, in the longitudinal arm, higher UFC levels.

**Figure 3.**
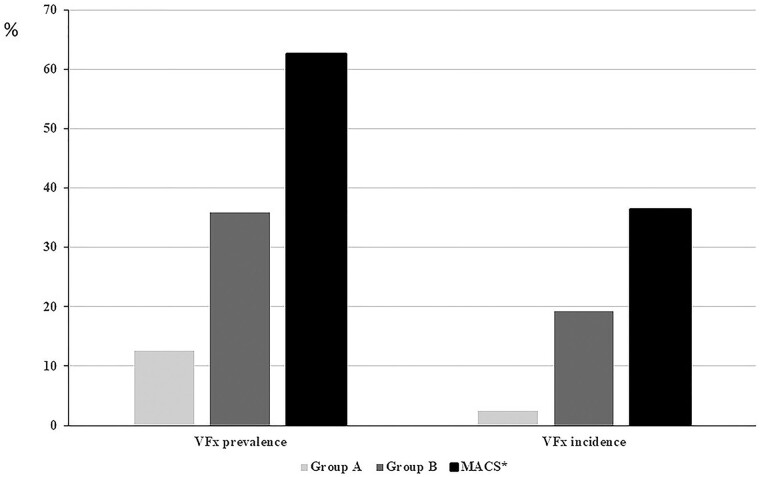
Comparison between the prevalence and incidence of vertebral fractures (VFx) in patients from group A, group B, and from a historical cohort of patients with mild autonomous cortisol secretion (MACS). Data are from [6].

The comparison between the prevalence and incidence of VFx in patients with MACS reported in our previous study [6] (62.6% and 36.4%, respectively) with those reported in patients from group B (35.9% and 19.3%, respectively) and from group A (12.6% and 3.6%, respectively) in the present study is of interest. Indeed, this comparison (Fig. 3) suggests that a decreasing trend exists in terms of bone damage from patients with MACS to patients with NFAI and F-1mgDST greater than or equal to 1.2 μg/dL (33 nmol/L) to patients with F-1mgDST less than 1.2 μg/dL (33 nmol/L).

From a clinical point of view, these data point out the need to evaluate AI patients by performing vertebral morphometry in addition to the BMD determination as already suggested by several authors for the management of patients with glucocorticoid-induced osteoporosis [33]. In particular, these data suggest that in NFAI patients with F-1mgDST greater than or equal to 1.2 µg/dL (33 nmol/L), it could be useful to perform a vertebral morphometry both at baseline and after a 24-month follow-up and to rely on F-1mgDST levels for monitoring possible cortisol hypersecretion and its possible deleterious effect on bone. Furthermore, we believe that, nowadays, data are too scarce to suggest adrenalectomy in patients with AI and F-1mgDST greater than 1.2 μg/dL (33 nmol/L). On the other hand, it could be conceivable to screen these patients for morphometric VFx and to consider antifracture therapy in the presence of a morphometric VFx and/or in the presence of a clearly reduced BMD even in the absence of osteoporotic values (ie, T-score BMD < −2.0).

The present study has some limits. First, its retrospective design does not exclude the fact that possible unknown confounding factors could have biased the results. Second, HPA axis activity parameters were measured only once. Since in some patients with AI cortisol secretion may fluctuate, measuring cortisol secretion only once could not be representative of the HPA axis activity over time in some patients with NFAI. Third, the sample size, particularly of the longitudinal arm, was probably too small to evaluate the independent association of VFx incidence with the presence of F-1mgDST greater than or equal to 1.2 µg/dL (33 nmol/L) and in general to draw firm conclusions. Fourth, control individuals were not investigated regarding AI presence for ethical reasons and the data regarding the degree of cortisol secretion were been collected. Therefore, although all control participants had F-1mgDST levels of less than 1.8 µg/dL (50 nmol/L), the possible effect of a different degree of cortisol secretion on bone health could not be investigated in controls. Fifth, we did not measure bone turnover markers and midnight salivary cortisol, which could have been informative. However, we should consider that the clinical use of bone turnover markers in the setting of glucocorticoid-induced osteoporosis is still a matter of debate [34] and that the determination of salivary cortisol at midnight is not of the utmost importance in the setting of mild hypercortisolism [35]. Similarly, the lack of a measurement of dexamethasone levels is an additional study limitation. Finally, we do not have data on family history for bone fragility, smoking habits, and physical activity, three important factors influencing bone health, which could have been highly informative.

Notwithstanding these aspects, the present study is of interest since it shows for the first time that NFAI patients with F-1mgDST greater than or equal to 1.2 µg/dL (33 nmol/L) have an increased prevalence and incidence of fragility fracture. Although they should be taken with caution, these data suggest the need for large longitudinal studies aimed to confirm whether NFAI patients with F-1mgDST greater than or equal to 1.2 µg/dL (33 nmol/L) may have relatively increased autonomous cortisol secretion. If these data were confirmed in another set of patients, they would change the management of patients with AI, since, as already suggested by others [36], the term *NFAI* could be considered inadequate in some AI patients so far defined as having a “nonfunctioning” adrenal tumor.

## Data Availability

All data sets generated during and/or analyzed during the present study are not publicly available but are available from the corresponding author on reasonable request.

## Abbreviations

ACTH: adrenocorticotropin
AI: adrenal incidentaloma
BMD: bone mineral density
BMI: body mass index
F-1mgDST: 1-mg overnight dexamethasone suppression test
FN: femoral neck
Fx: fractures
HPA: hypothalamic-pituitary-adrenal axis
LS: lumbar spine
MACS: mild autonomous cortisol secretion
NFAI: nonfunctioning adrenal incidentaloma
nonvertebral Fx: fragility fractures in other skeletal sites (femur, humerus, and wrist)
ROC: receiver operating characteristic
T2D: type 2 diabetes mellitus
UFC: urinary free cortisol
VFx: vertebral fractures
